# Association between polymorphisms of collagen genes and susceptibility to intervertebral disc degeneration: a meta-analysis

**DOI:** 10.1186/s13018-021-02724-8

**Published:** 2021-10-18

**Authors:** Guohui Xie, Chunhong Liang, Honglin Yu, Qin Zhang

**Affiliations:** Department of Spine Surgery, Yuncheng Central Hospital, Shanxi Medical University, No. 3690, Hedong East Street, Yanhu District, Yuncheng, 044000 Shanxi Province China

**Keywords:** COL9A2, COL9A3, COL1A1, COL11A1, Intervertebral disc degeneration, Meta-analysis

## Abstract

**Background:**

Collagens are important structural components of intervertebral disc. A number of studies have been performed for association between polymorphisms of collagen genes and risk of intervertebral disc degeneration (IVDD) but yielded inconsistent results. Here, we performed a meta-analysis to investigate the association of collagen IX alpha 2 (COL9A2) Trp2, collagen IX alpha 3 (COL9A3) Trp3, collagen I alpha 1 (COL1A1) Sp1 and collagen XI alpha 1 (COL11A1) C4603T polymorphisms with susceptibility to IVDD.

**Method:**

Eligible studies were retrieved by searching MEDLINE, EMBASE, Web of Science prior to 31 March, 2021. Odds ratio (OR) and corresponding 95% confidence interval (CI) were calculated for association strength.

**Results:**

A total of 28 eligible studies (31 datasets comprising 5497 cases and 5335 controls) were included. COL9A2 Trp2 carriers had an increased risk of IVDD than non-carriers in overall population (OR = 1.43, 95% CI 0.99–2.06, *P* = 0.058), which did not reach statistical significance. However, Trp2 carriers had 2.62-fold (95% CI 1.15–6.01, *P* = 0.022) risk than non-carriers in Caucasians. COL9A3 Trp3 was not associated with IVDD risk (OR = 1.28, 95% CI 0.81–2.02, *P* = 0.299). T allele and TT genotype of COL1A1 Sp1 (+ 1245G > T) were correlated with increased risk of IVDD. Significant associations were found between COL11A1 C4603T and IVDD risk under allelic (OR = 1.33, 95% CI 1.20–1.48), dominant (OR = 1.45, 95% CI 1.26–1.67), recessive (OR = 1.55, 95% CI 1.21–1.98) and homozygote model (OR = 1.81, 95% CI 1.40–2.34).

**Conclusions:**

COL1A1 Sp1 and COL11A1 C4603T polymorphism are associated with IVDD risk while the predictive roles of collagen IX gene Trp2/3 need verification in more large-scale studies.

**Supplementary Information:**

The online version contains supplementary material available at 10.1186/s13018-021-02724-8.

## Background

Intervertebral disc degeneration (IVDD) is a prevalent health problem worldwide and mainly contributes to neck and low back pain, disc herniation and sciatica [1]. The aetiology and pathogenesis of IVDD are complicated and have not been fully elucidated. Environmental factors such as mechanical forces, smoking, sex, age and body mass index (BMI) may partially contribute to the IVDD development [2]. However, twin studies identified genetic factors as the main determinants of IVDD and yielded a heritability estimate that was up to 74% [3, 4]. Genetic association studies have shed light on the single nucleotide polymorphisms (SNPs) associated with IVDD susceptibility [5]. To date, numerous polymorphisms in genes encoding collagens [6], carbohydrate sulfotransferase (CHST) [7], interleukins [8], matrix metalloproteinases (MMP) [9], apoptosis-inducing ligand (TRAIL) [10] and growth differentiation factors (GDF) [11] have been investigated. These genes can be functionally incorporated into categories of intervertebral disc structure, structural support, cytokines, extracellular matrix-degrading enzymes, apoptotic factors, growth factors [5], each of which plays a different role in the development of disc degeneration.

Intervertebral disc is composed of the outer annulus fibrosis region (AF) and the central nucleus pulposus (NP). Collagens are important components of extracellular matrix (ECM) of intervertebral disc and are detected in AF and NP in large amounts [12]. Specifically, type I, IX and XI collages have attracted much attentions. Collagen I is the primary type of collagen in AF that is responsible for retaining NP and distributing the compressive load [13]. Two genes, collagen type 1 alpha 1 (COL1A1) and alpha 2 (COL1A2), encode the α1 and α2 chain of collagen I, respectively. Previous studies identified a correlation of a Sp1-binding site polymorphism of COL1A1 (+ 1245G > T, rs1800012) with IVDD risk that carriers of TT genotype were more vulnerable to disc degeneration [14, 15]. Collagen IX is made up of α1, α2 and α3 chains, which were encoded by collagen type 9 alpha 1 (COL9A1), alpha 2 (COL9A2) and alpha 3 (COL9A3) genes, respectively [16]. Unlike the other abundantly expressed constitutive collagens, collagen IX increased intervertebral disc strength by connecting various types of constitutive collagens together and linking collagens with non-collagen components of ECM [5, 13]. A sequence variation of COL9A2 resulting in an amino acid substitution from Gln to Trp at the 326th residue (rs137853213, Trp2) was identified in IVDD patients but not in normal controls [17, 18]. Another substitution from Arg to Trp at the 103^rd^ residue (rs61734651, Trp3) of COL9A3 was found associated with an increased risk of IVDD [19]. Collagen XI is a cartilage-specific ECM protein expressed in both AF and NP and participates in the formation of cartilage fibrils with other collagens, particularly collagen II and collagen IX [20]. A common missense variant (c.C4603T;p.Ser1535Pro, rs1676486) of COL11A1 encoding the α1 chain of collage XI was identified as a risk factor of IVDD in Japanese and Chinese populations [21, 22]. These putatively functional polymorphisms may participate in the development of disc degeneration through altering the gene expression pattern or interaction with other collagens.

However, more recent studies found a lack of association in independent populations, implying the association between these polymorphisms (COL1A1 Sp1, COL9A2 Trp2, COL9A3 Trp3, COL11A1 C4603T) and IVDD predisposition was still in controversy [23–26]. Here, we performed a systematic review and meta-analysis for these functional SNPs in collagen genes with IVDD susceptibility.

## Methods

### Literature search

We performed the present meta-analysis in compliance with the Preferred Reporting Items for Systematic Reviews and Meta-Analyses (PRISMA). The PRISMA checklist can be found in Additional file 1. Relevant studies evaluating the associations between polymorphisms of collagen genes and susceptibility to disc degeneration were retrieved by searching MEDLINE, EMBASE, Web of Science prior to 31 March, 2021, using the following terms: (disc degeneration OR degenerative disc disease OR lumbar disc disease OR intervertebral disc disease lumbar disc herniation OR LDD OR IVDD) AND (collagen OR COL9A2 OR COL9A3 OR COL11A1 OR COL1A1) AND (SNP OR polymorphism OR variant OR variation). There was no language restriction. The reference lists of eligible articles were further screened for additional candidate studies.

### Inclusion and exclusion criteria

Eligible studies should follow these criteria: (1) investigated the relationships of COL9A2 Trp2 (Gln326Trp, rs137853213), COL9A3 Trp3 (Arg103Trp, rs61734651), COL1A1 Sp1 (rs1800012) or COL11A1 C4603T (Ser1535Pro, rs1676486) with disc degeneration, (2) was a case–control or cohort study, (3) provided distributions of genotype and/or allele in both case and control groups. Case reports, reviews, meta-analyses and studies without full-text or available genotype data were excluded. If the genotype frequency of control group was not in Hardy–Weinberg Equilibrium (HWE *P* < 0.05), the study was also excluded. For repeated publications, only the most complete or recent one was included.

### Data extraction and quality assessment

The following items of each eligible study were extracted: first author, year of publication, country, ethnicity, disease, diagnostic criteria, age, per cent of male, genotyping method, source of control, sample size, genotype and allele distributions. The quality of eligible study was assessed by using Newcastle–Ottawa Scale (NOS). The total score of NOS ranged from 0 to 9, and ≥ 7 scores indicated high quality. The literature search, selection of eligible studies, data extraction and quality assessment were performed by two independent investigators, and discrepancies were resolved by discussion with a third investigator.

### Statistical analysis

Pooled odds ratio (OR) and corresponding 95% confidence interval (95% CI) were calculated for the association strength between polymorphism and risk of disc degeneration. The between-study heterogeneity was assessed by *I*^2^ and *Q* test. *I*^2^ < 50% and *P* value for *Q* test > 0.10 indicated no obvious heterogeneity, and then, a fixed effect model was used for pooled analysis. Otherwise, there was significant heterogeneity and a random effect model was used. Since the homozygous variants of COL9A2 Trp2 and COL9A3 Trp3 were both in low frequency, we only compared the risk of Trp2 or Trp3 carriers to that of non-carriers. For COL1A1 and COL11A1 polymorphisms, the associations were analysed under four genetic models: allelic model, dominant model, recessive model and homozygote model. Sensitivity analysis was performed to evaluate the robustness of meta-analysis and potential source of heterogeneity by excluding one study at a time. Funnel plot and Egger’s test were conducted for publication bias assessment. STATA 12.0 (Stata Corporation, TX, US) was used for statistical analysis. A *P* value < 0.05 indicated statistical significance.

## Results

### Characteristics of studies included in the meta-analysis

**Fig. 1:**
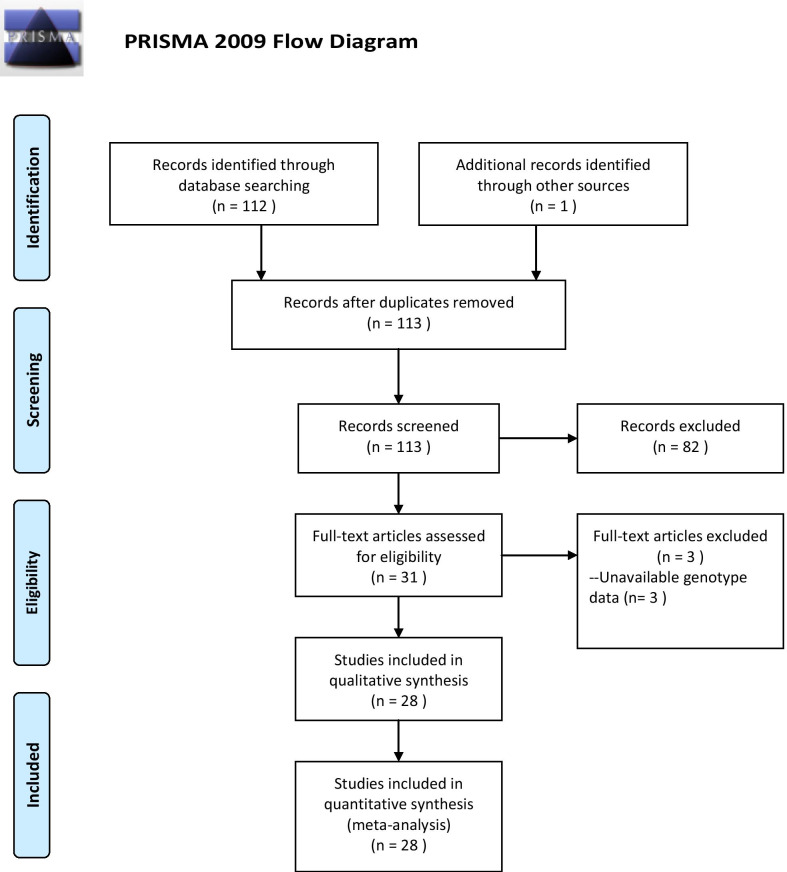
Flow diagram of the literature search and selection

A total of 31 relevant publications investigating the correlation between collagen polymorphisms and disc degeneration susceptibility were obtained by literature search and selection. We furtherly excluded 3 studies because of unavailable genotype data [27–29]. Mio’s study had 3 independent datasets [22] and Koyoma’s study had two datasets [24], and then, each dataset was individually included in the quantitative analysis. Therefore, 28 studies (31 datasets) comprising 5497 cases and 5335 controls were finally included in our meta-analysis [14, 15, 17–19, 21–26, 30–46]. The flow diagram of the literature search is shown in Fig. 1. Fifteen studies (2292 cases and 2089 controls) investigated the correlation between COL9A2 Trp2 and disc degeneration susceptibility, 13 studies (1623 cases and 1606 controls) for COL9A3 Trp3, 4 studies (310 cases and 812 controls) for COL1A1 sp1, and 5 studies (8 datasets, 1817 cases and 1728 controls) for COL11A1 C4603T. According to NOS, all studies were of high quality (NOS scores ≥ 7). The baseline characteristics of all eligible studies are summarized in Table 1. The numbers of Trp2 or Trp3 carriers and non-carriers are listed in Table 2, while the genotype and allele distributions of COL1A1 Sp1 polymorphism and COL11A1 C4603T are listed in Table 3. The genotype distributions of control group of COL1A1 sp1 and COL11A1 C4603T were all in Hardy–Weinberg Equilibrium [47] (*P* > 0.05, Table 3).

**Table 1:** Baseline characteristics of the studies included in the meta-analysis

References	Country	Conditions	Sample size		Genotyping method	Polymorphism	NOS
			Case	Control			
Annunen [18]	Finland	IVDD	314	202	CSGE, sequencing	Trp2	8
Paassilta [19]	Finland	LDD	171	321	CSGE, sequencing	Trp3	7
Noponen-Hietala [46]	Finland	LDD	29	56	CSGE, sequencing	Trp2, Trp3	7
Pluijm [15]	Netherland	IVDD	118	375	PCR–RFLP	Sp1	8
Kales [44]	Greece	IVDD	105	102	PCR-pyrosequencing	Trp2, Trp3	8
Matsui [17]	America	IVDD	97	10	Sequencing	Trp2, Trp3	7
Tilkeridis [43]	Greece	LDD	24	12	Sequencing	Sp1	7
Jim [41]	China	IVDD	514	290	CSGE, sequencing	Trp2, Trp3	8
Solovieva [42]	Finland	LDD	77	77	CSGE, sequencing	Trp2, Trp3	8
Seki [40]	Japan	LDD	470	658	Sequencing, TaqMan	Trp2	8
Mio [22]	Japan	LDD	881	841	TaqMan	C4603T	8
Song [38]	China	IVDD	125	125	SNPstream	Trp2	7
Hyun [37]	Korea	LDD	205	101	Sequencing	Trp2	7
Zhu [36]	America	IVDD	25	7	NR	Trp2, Trp3	7
Eskola [39]	Denmark	LDD	66	154	SNaPshot	Trp3	8
Kelempisioti [35]	Finland	IVDD	150	246	SNaPshot	Trp2, Trp3	8
Lim [45]	Singapore	LDD	34	20	Sequencing, TaqMan	Trp2, Trp3	7
Koyama [34]	Japan	IVDD	44	55	TaqMan	C4603T	8
Omair [33]	Norway	LDD	146	188	MassARRAY	Trp3, C4603T	8
Rathod [32]	India	IVDD	100	100	Real-time PCR	Trp2, Trp3	7
Lin [14]	China	IVDD	375	118	PCR–RFLP	Sp1	8
Anjankar [26]	India	IVDD	50	50	PCR	Sp1	8
Bagheri [25]	Iran	IVDD	108	57	Tetraplex PCR	Trp2, Trp3	7
Liu [21]	China	LDD	657	532	TaqMan	C4603T	8
Koyama [24]	Japan	IVDD	101	114	TaqMan	C4603T	8
Hanaei [23]	Iran	IVDD	96	94	TaqMan	Trp2	7

IVDD, intervertebral disc degeneration; LDD, lumbar disc degeneration; CSGE, conformation-sensitive gel electrophoresis; PCR, polymerase chain reaction; RFLP, restriction fragment length polymorphism; NOS, Newcastle–Ottawa Scale

**Table 2:** Number of collagen IX Trp2/3 carriers and non-carriers of the studies included in the meta-analysis

Polymorphism	Ethnicity	Case group		Control group	
		Carriers	Non-carriers	Carriers	Non-carriers
*COL9A2 Trp2, rs137853213*					
Annunen [18]	Caucasian	6	151	0	174
Noponen-Hietala [46]	Caucasian	1	28	0	56
Kales [44]	Caucasian	0	105	0	102
Matsui [17]	Mixed	4	93	0	10
Jim [41]	Asian	108	406	52	238
Solovieva [42]	Caucasian	3	77	0	55
Seki [40]	Asian	100	370	150	504
Song [38]	Asian	30	95	25	100
Hyun [37]	Asian	50	155	23	76
Zhu [36]	Mixed	1	24	0	7
Kelempisioti [35]	Caucasian	6	144	8	238
Lim [45]	Asian	4	30	3	17
Rathod [32]	Asian	57	43	17	83
Bagheri [25]	Asian	34	74	13	44
Hanaei [23]	Asian	37	56	41	53
*COL9A3 Trp3, rs61734651*					
Paassilta [19]	Caucasian	40	131	30	291
Noponen-Hietala [46]	Caucasian	4	26	0	56
Kales [44]	Caucasian	9	96	5	97
Matsui [17]	Mixed	7	90	0	10
Jim [41]	Asian	0	514	0	290
Solovieva [42]	Caucasian	15	62	8	47
Zhu [36]	Mixed	5	20	1	6
Eskola [39]	Caucasian	9	57	31	123
Kelempisioti [35]	Caucasian	22	128	52	194
Lim [45]	Asian	0	34	0	20
Omair [33]	Caucasian	17	129	23	165
Rathod [32]	Asian	5	95	7	93
Bagheri [25]	Asian	29	79	10	47

**Table 3:** Genotype and allele distributions of COL1A1 and COL11A1 polymorphisms

Polymorphism	Ethnicity	Case group					Control group					
		Genotype			Allele		Genotype			Allele		HWE
COL1A1 Sp1, rs1800012		GG	GT	TT	G	T	GG	GT	TT	G	T	
Pluijm [15]	Caucasian	82	28	8	192	44	264	102	9	630	120	0.818
Tilkeridis [43]	Caucasian	6	10	8	22	26	4	8	0	16	8	0.083
Lin [14]	Asian	82	26	10	190	46	264	102	9	630	120	0.818
Anjankar [26]	Asian	38	10	2	86	14	39	10	1	88	12	0.708
COL11A1 C4603T, rs1676486		CC	CT	TT	C	T	CC	CT	TT	C	T	
Mio [22] dataset 1	Asian	85	86	17	256	120	99	67	13	265	93	0.721
Mio [22] dataset 2	Asian	149	163	47	461	257	154	108	21	416	150	0.732
Mio [22] dataset 3	Asian	156	144	34	456	212	200	150	26	550	202	0.767
Koyama [34]	Asian	29	9	6	67	21	33	18	4	84	26	0.488
Omair [33]	Caucasian	–	–	–	225	67	–	–	–	293	81	–
Liu [21]	Asian	263	316	68	842	452	272	221	39	765	299	0.518
Koyama [24] dataset 1	Asian	17	20	8	54	36	24	22	1	70	24	0.113
Koyama [24] dataset 2	Asian	23	28	3	74	34	31	29	9	91	47	0.593

HWE, Hardy–Weinberg equilibrium

### Association between COL9A2 Trp2 and IVDD risk

**Fig. 2:**
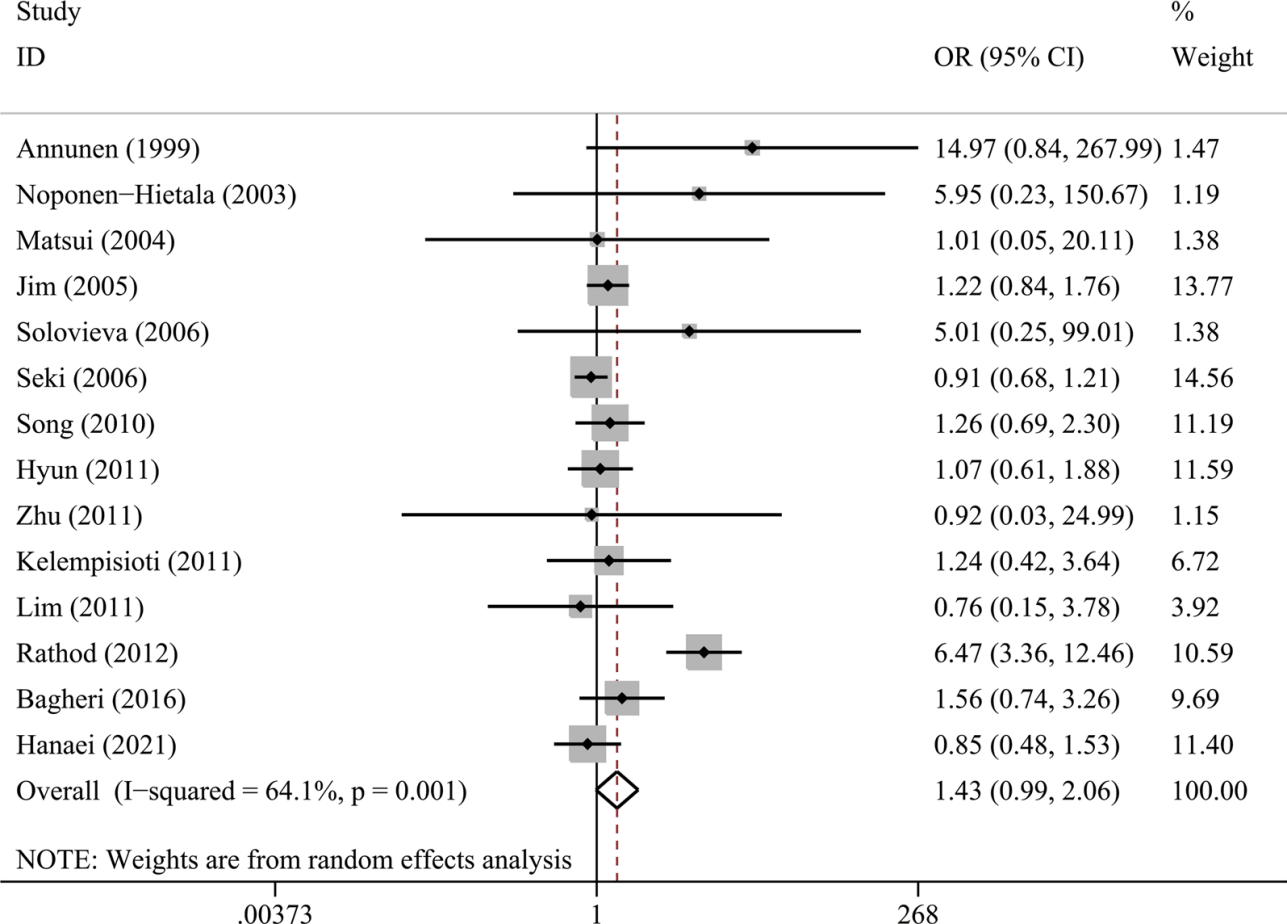
Forest plot for association between COL9A2 Trp2 and risk of intervertebral disc degeneration

We excluded Kales SN’s study [44] since no Trp2 was found in the participants and pooled the rest 14 studies comprising 2817 cases and 1987 controls together (Table 4). Meta-analysis using a random effect model demonstrated an increased risk of disc degeneration in COL9A2 Trp2 carriers compared to non-carriers (OR = 1.43, 95% CI 0.99–2.06, *I*^2^ = 64.1%, Fig. 1). However, the association did not reach statistical significance (*P* = 0.058). Subgroup analysis in Caucasian population showed that Trp2 carriers had a significantly higher risk compared to non-carriers (OR = 2.62, 95% CI 1.15–6.01, *P* = 0.022). In the subgroups of Asian population and mixed ethnical population, no significant association was found between Trp2 and IVDD predisposition. Three studies provided genotype data of male subgroup [32, 42, 45], and meta-analysis showed higher disc degeneration risk in males with Trp2 variant (OR = 3.00, 95% CI 1.57–5.74). However, the sample size is relatively small and the results need verification in large-scale populations (Fig. 2).

**Table 4:** Association between Trp2/3 polymorphisms and disc degeneration susceptibility

Polymorphism	No. of studies	Sample size (case/control)	Pooled effect size		Heterogeneity	
			OR (95% CI)	*P*	*I*^2^ (%)	*P*
*COL9A2 Trp2, rs137853213*						
Overall	14	2187/1987	1.43 (0.99–2.06)	0.058	64.1	< 0.001
*Ethnicity*						
Caucasian	4	416/531	2.62 (1.15–6.01)	0.022	18.6	0.298
Asian	8	1649/1439	1.35 (0.89–2.04)	0.154	77.7	< 0.001
Mixed	2	122/17	0.97 (0.11–8.86)	0.977	0	0.966
*Gender*						
Male	3	179/142	3.00 (1.57–5.74)	0.001	41.2	0.182
*COL9A3 Trp3, rs61734651*						
Overall	11	1075/1296	1.28 (0.81–2.02)	0.299	60.5	0.005
*Ethnicity*						
Caucasian	7	745/1122	1.31 (0.71–2.41)	0.390	74.7	< 0.001
Asian	2	208/157	1.30 (0.68–2.50)	0.422	34.7	0.216
Mixed	2	122/17	1.60 (0.26–9.88)	0.615	0	0.938
*Gender*						
Male	6	286/268	1.12 (0.42–2.97)	0.827	55.7	0.046
Female	5	195/207	1.11 (0.62–2.01)	0.725	0	0.802

### Association between COL9A3 Trp3 and IVDD risk

**Fig. 3:**
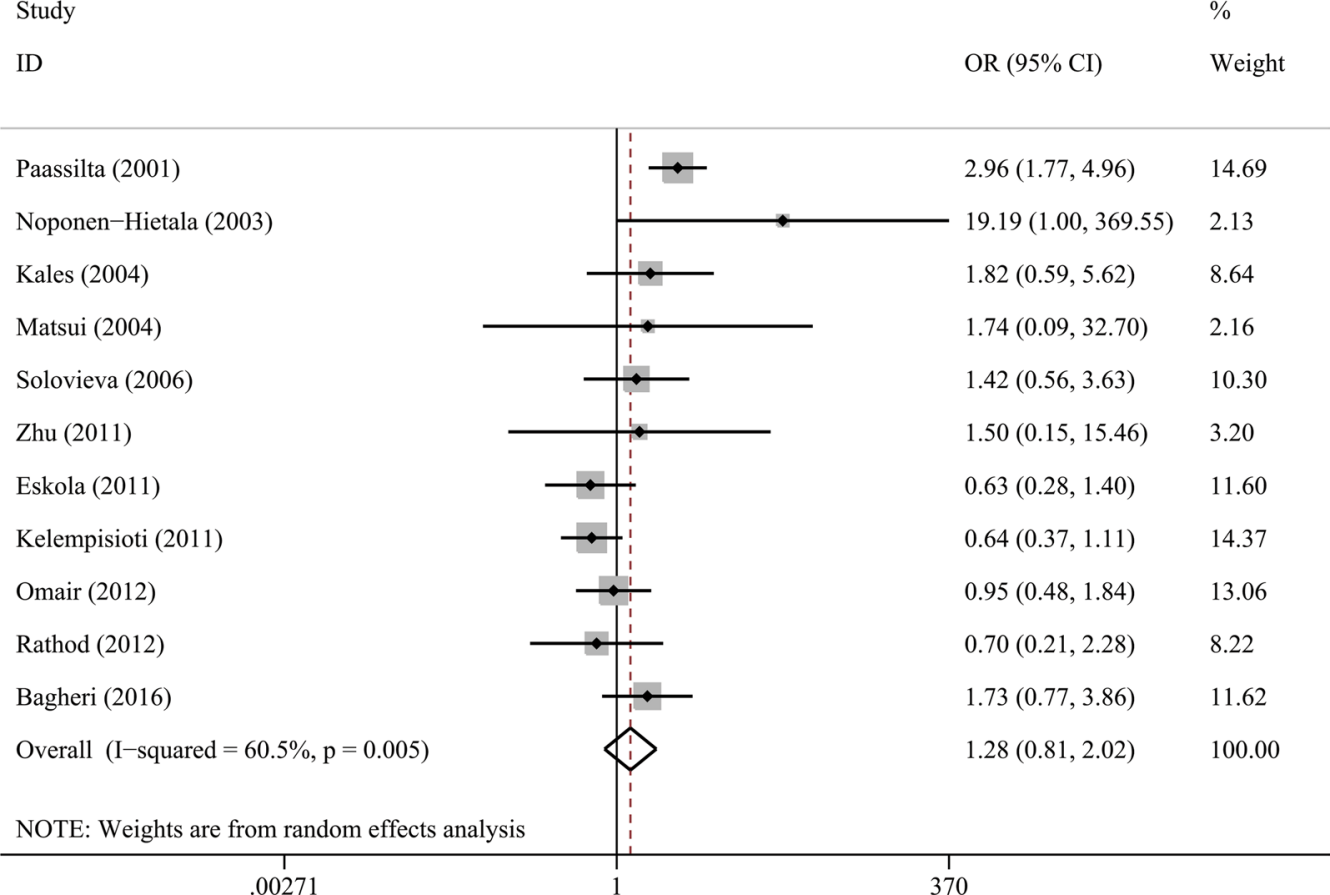
Forest plot for association between COL9A3 Trp3 and risk of intervertebral disc degeneration

The Trp3 variant was not found in two studies with 548 cases and 310 controls from Asian populations [41, 45]. Thus, 11 studies with 1075 cases and 1296 controls were finally quantitatively synthesized (Table 4). Meta-analysis using a random effect model showed that Trp3 was not significantly associated with risk of disc degeneration (OR = 1.28, 95% CI 0.81–2.02, *P* = 0.299, Fig. 3). Subgroup analyses stratified by ethnicity and gender were performed but no significant associations were found.

### Association between COL1A1 Sp1 polymorphism and IVDD risk

**Fig. 4:**
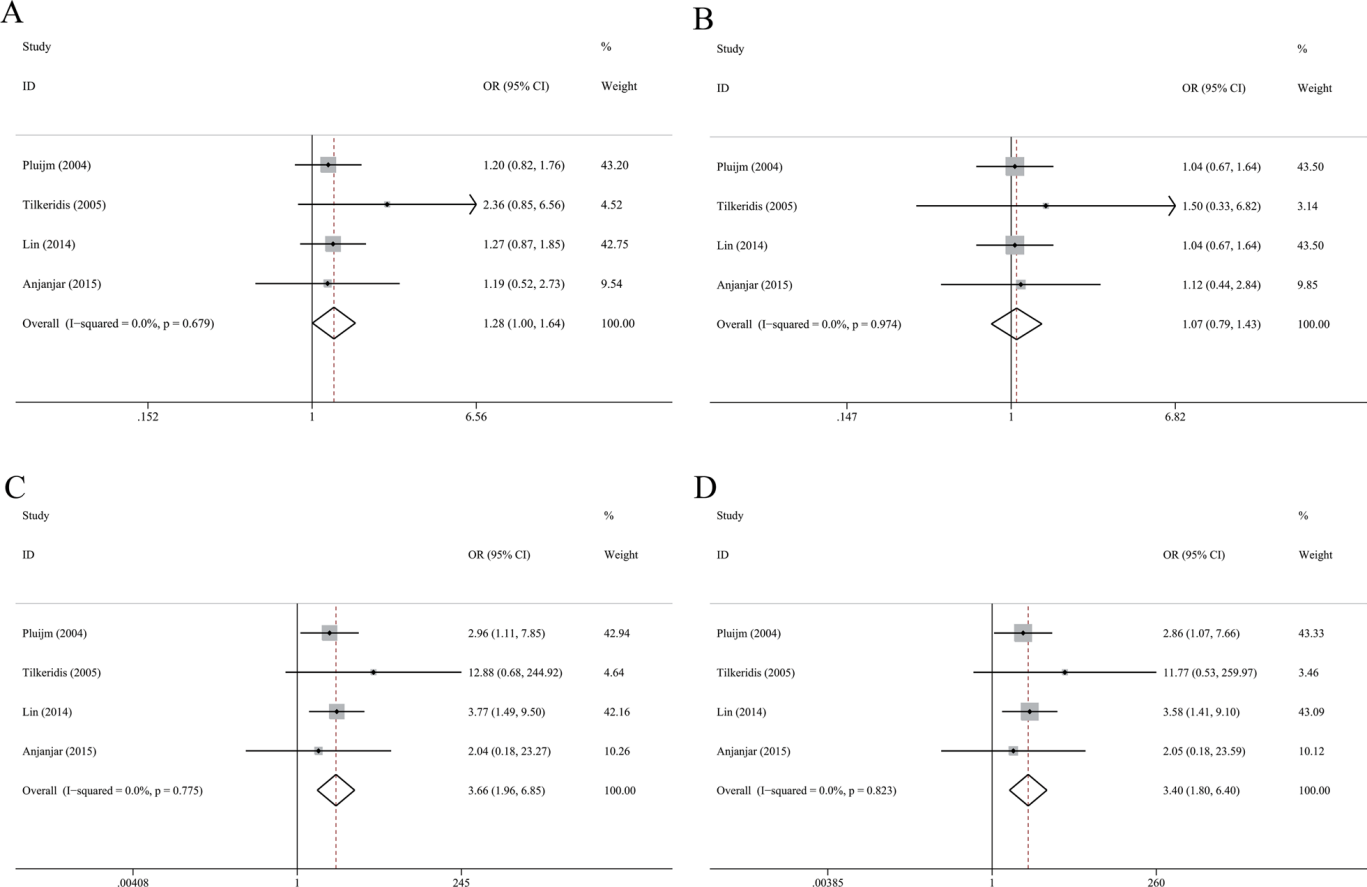
Forest plot for association between COL1A1 Sp1 and risk of intervertebral disc degeneration in allelic (**A**), dominant (**B**), recessive (**C**) and homozygote (**D**) models

We included 4 eligible studies comprising 310 cases and 812 controls in the meta-analysis and used the fixed effect model because of no between-study heterogeneity (Table 5). Sp1 polymorphism was correlated with increased risk of disc degeneration under allelic model (T vs. G: OR = 1.28, 95% CI 1.00–1.64, *P* = 0.047, Fig. 4A), recessive model (TT vs. GG + GT: OR = 3.66, 95% CI 1.96–6.85, *P* < 0.001, Fig. 4C), and homozygote model (TT vs. GG: OR = 3.40, 95% CI 1.80–6.40, *P* < 0.001, Fig. 4D). However, there was no significant association under dominant model (TT + GT vs. GG, Fig. 4B).

**Table 5:** Association between polymorphisms of COL1A1, COL11A1 and disc generation susceptibility

Polymorphism	No. of studies	Pooled effect size		Heterogeneity	
		OR (95% CI)	*P*	*I*^2^ (%)	*P*
*COL1A1 Sp1, rs1800012*					
T vs G	4	1.28 (1.00–1.64)	0.047	0	0.679
TT + GT vs GG	4	1.06 (0.79–1.43)	0.671	0	0.974
TT vs GG + GT	4	3.66 (1.96–6.85)	< 0.001	0	0.775
TT vs GG	4	3.40 (1.80–6.40)	< 0.001	0	0.823
*COL11A1 C4603T, rs1676486*					
T vs C	8	1.33 (1.20–1.48)	< 0.001	4.0	0.399
TT + CT vs CC	7	1.45 (1.26–1.67)	< 0.001	0	0.600
TT vs CC + CT	7	1.55 (1.21–1.98)	0.001	23.3	0.251
TT vs CC	7	1.81 (1.40–2.34)	< 0.001	19.7	0.279

### Association between COL11A1 C4603T and IVDD risk

**Fig. 5:**
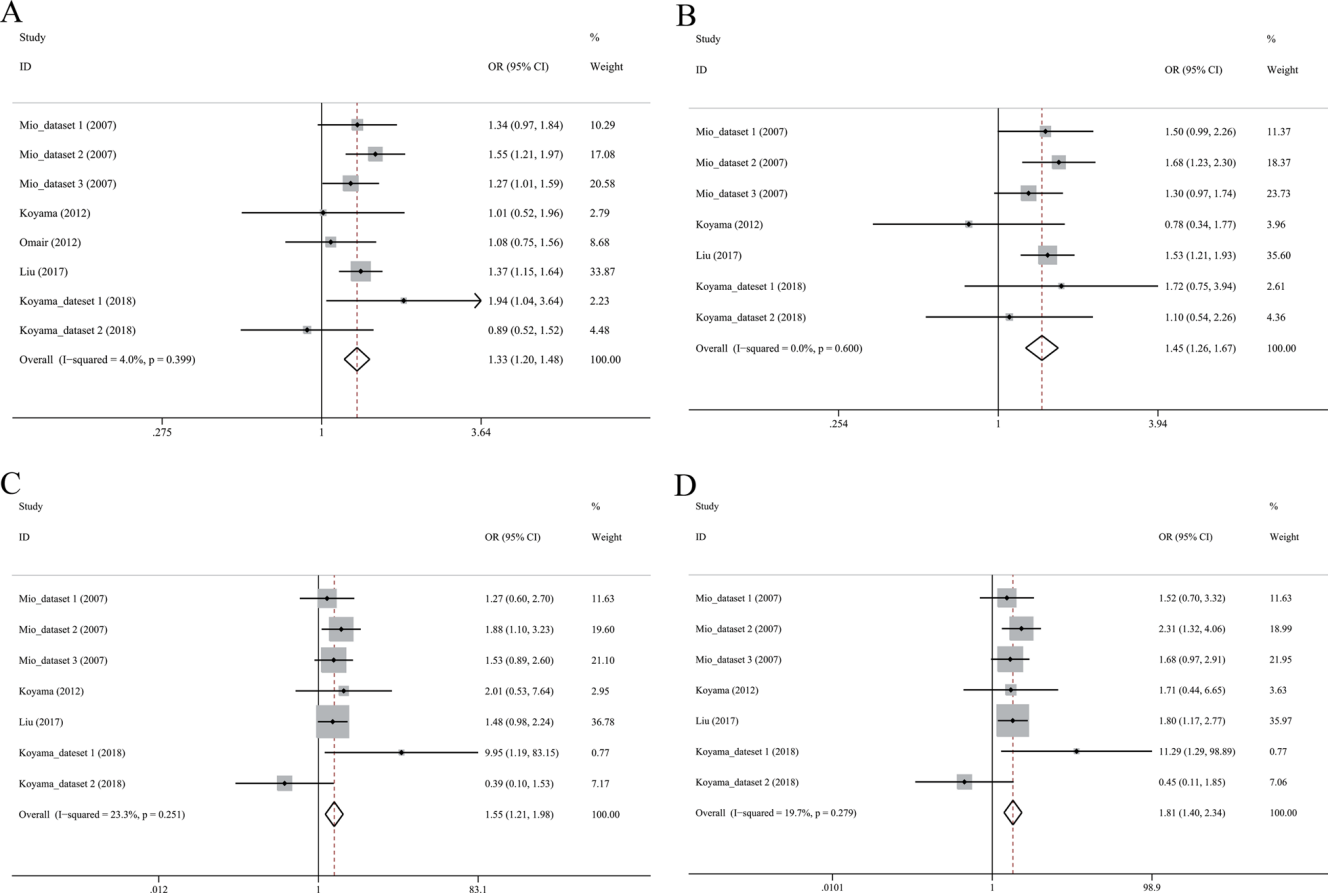
Forest plot for association between COL11A1 C4603T and risk of intervertebral disc degeneration in allelic (**A**), dominant (**B**), recessive (**C**) and homozygote (**D**) models

Eight datasets with 1817 cases and 1728 controls were included. There was no obvious between-study heterogeneity, and a fixed effect model was used (Table 5). T allele was significantly associated with an increased risk of disc degeneration (OR = 1.33, 95% CI 1.20–1.48, *P* < 0.001, Fig. 5A). Under dominant model, carriers of TT or CT genotype had a 1.45-fold (95% CI 1.26–1.87, *P* < 0.001, Fig. 5B) risk of disc degeneration compared to CC genotype. Carriers of TT genotype were more susceptible to disc degeneration when compared to CC or CT genotype (OR = 1.55, 95% CI 1.21–1.98, *P* = 0.001, Fig. 5C) and CC genotype carriers (OR = 1.81, 95% CI 1.40–2.34, *P* < 0.001, Fig. 5D).

### Sensitivity analysis and publication bias

**Fig. 6:**
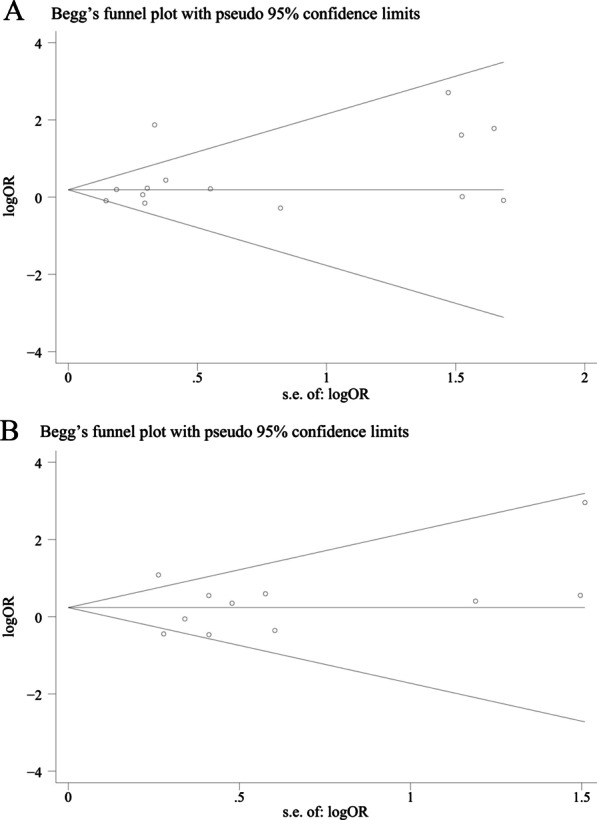
Funnel plots for COL9A2 Trp2 (**A**) and COL9A3 Trp3 (**B**)

Sensitivity analysis showed that Rathod’s study [32] was the main source of heterogeneity for Trp2 analysis. After excluding this study, the heterogeneity reduced from 64.1% to 0, and Trp2 was not associated with disc degeneration susceptibility in overall population (OR = 1.09, 95% CI = 0.92–1.30, *P* = 0.317). The funnel plots were all symmetric (Fig. 6) and *P* values for Egger’s test were > 0.05, indicating no evidence of publication bias.

## Discussion

The present meta-analysis, incorporating 5497 cases and 5335 controls from 28 studies, demonstrated significant correlations of COL1A1 sp1 and COL11A1 C4603T polymorphisms with susceptibility to IVDD. Furthermore, the meta-analysis revealed that COL9A2 Trp2 was associated with IVDD predisposition in Caucasian population and that COL9A3 Trp3 had no correlation with IVDD risk. The results indicated the important role of collagens in the development of disc degeneration.

Collagen type IX plays a connective role in creating cross-links between various types of collagens in intervertebral disc [5, 13]. Mutations or polymorphisms may cause dysfunction of collagen IX and predispose carriers to disc degeneration [48]. Transgenic mice with mutations in Col9a1 encoding the α1 chain of collagen IX developed various forms of degenerative changes in spine and joints [49]. The Trp2 allele, an amino acid change from Gln to Trp, is the most common polymorphism in COL9A2 that encodes the α2 chain of collagen IX [16]. The Trp3 allele represents a substitution of Arg by Trp in COL9A3 encoding the α3 chain of collagen IX [16]. Both Trp alleles are hydrophobic and cause increased insolubility of collagen IX, which affect the interactions between collagens and ECM components and influence the disc mechanics resisting against compressive load [48]. Aladin DM et al. measured the swelling pressure and compressive modulus in Trp2 positive and negative non-degenerated discs [50]. They found these indicators were significantly lowers in Trp2 + samples than in Trp2- samples, suggesting that Trp2 may diminish the mechanical properties of disc [50].

However, our meta-analysis did not find significant associations between Trp2 allele and disc degeneration risk in overall populations, which may be caused by varied allele frequencies in different populations. In Finish population of European ancestry, Trp2 allele is only found at a low frequency in disc degeneration patients but absent in normal controls [17, 18, 42], implying a disease-causing role of this variant. In contrast, Trp2 allele is common in East Asian countries including China, Japan, Korea and Singapore, and does not differ in frequency between patients and normal controls [37, 38, 40, 41, 45]. Subgroup analysis by ethnicity showed that Trp2 was significantly associated with IVDD susceptibility in Caucasians but not in Asians. Despite lacking association in Asians, Jim et al. found a 2.4-fold increase in IVDD risk of Trp2 positive individuals aged 30–39 years in a large cohort of Chinese population, indicating that Trp2 is an age-dependent risk factor [41]. Thus, we speculate that interactions between environment factors and Trp2 allele may contribute to disc degeneration development in Asians. This is the first meta-analysis for COL9A2 Trp2 (rs137853213) with IVDD susceptibility. Previous meta-analyses focusing on COL9A2 rs12077871, rs12722877 and rs7533552 polymorphisms revealed no significant associations with susceptibility to lumbar disc degeneration [51, 52].

We also observed divergent frequency of Trp3 allele in Caucasians and Asians. Contrary to Trp2, Trp3 allele is frequent in populations of Caucasian ancestry but totally absent from Chinese population [41]. Overall analysis and subgroup analyses stratified by ethnicity and gender reveal that Trp3 allele is not a risk factor for disc degeneration, which is similar to previous meta-analyses [51, 53].

COL1A1 rs1800012 is located at a Sp1-binding site in intron 1 with a nucleotide change from guanine to thymine (G > T) [54]. The T allele has increased binding affinity with the transcription factor Sp1 and elevated expression of mRNA and encoded protein, leading to imbalanced ratio of two chains (α1/α2) of collagen I and instability of collagen fibres [55]. This polymorphism has been associated with several musculoskeletal traits, including low bone mineral, osteoporosis and osteoporotic fracture [56–58]. Our analysis showed that COL1A1 Sp1 polymorphism was also associated with susceptibility to IVDD and TT genotype conferred more than threefold risk to disc degeneration than GG genotype.

The present meta-analysis, having a larger sample size than the previous one [6], demonstrated that COL11A1 C4603T polymorphism was associated with IVDD susceptibility in a dosage-dependent manner (CT vs CC, OR = 1.39, 95% CI 1.20–1.61; TT vs CC, OR = 1.81, 95% CI 1.40–2.34). The transcript containing T allele degraded faster than the wildtype transcript, resulting in lower expression levels of mRNA and protein in intervertebral disc [22]. Compared to CC or CT genotype, the TT genotype carriers had remarkedly decreased COL11A1 mRNA expression in disc tissues and higher grade of severity of disc degeneration [21]. These findings suggest that T allele of COL11A1 C4603T polymorphism may increase IVDD susceptibility by reducing mRNA expression and the subsequent protein expression of COL11A1 in disc tissue.

Besides collagens, many factors also contribute to ECM structure feature and mechanical load distribution in intervertebral discs. Fibronectin, a core component of ECM with special spatial expression pattern in intervertebral discs, may help to organize the structure of discs [59]. TREK-1, encoding a potassium channel in response to mechanical and chemical stimuli, is found in NP and AF of intervertebral discs [60]. These findings indicate that the maintenance of normal structure and mechanical property of discs are important for prevention of IVDD. Although surgery has been proven to be effective, many biological strategies, for example, mesenchymal stem cells, growth factors and anticatabolic substances, are under investigation for potential clinical applications in prevention and management of IVDD [61, 62].

Our study has some limitations. Firstly, there was substantial heterogeneity for COL9A2 Trp2 and COL9A3 Trp3, which may be resulted from the difference in genetic background, definition of cases and controls, or occupations of participants. Thus, the results should be interpreted cautiously. Secondly, we failed to performed subgroup analyses stratified by age and occupations for all polymorphisms, and by gender and ethnicity for COL1A1 Sp1 and COL11A1 C4603T polymorphisms, to eliminate the influence of these confounders. Thirdly, the number of included studies and sample size for COL1A1 Sp1 was relatively small. Future studies with large sample sizes are warranted.

## Conclusions

In conclusion, COL1A1 Sp1 polymorphism and COL11A1 C4603T are markers of IVDD susceptibility, and interventions targeting these loci or modulating gene expression may help to prevent development and progression of IVDD. In addition, COL9A2 Trp2 is a risk factor of IVDD in Caucasian population but COL9A3 Trp3 was not correlated with IVDD susceptibility. More well-designed clinical trials with large sample size and performed in different ethnic populations are warranted in the future.

## Supplementary Information

**Additional file 1**. PRISMA 2020 checklist.

## Data Availability

The datasets used and/or analysed during the current study are available from the corresponding author on reasonable request.

## Abbreviations

COL9A2: Collagen IX alpha 2
COL9A3: Collagen IX alpha 3
COL1A1: Collagen I alpha 1
COL11A1: Collagen XI alpha 1
IVDD: Intervertebral disc degeneration
LDD: Lumbar disc degeneration
ECM: Extracellular matrix
NOS: Newcastle–Ottawa Scale
OR: Odds ratio
